# Piezoelectric Energy Harvester with Zigzag Root Section for Self-Powered Transformer Monitoring

**DOI:** 10.3390/mi16121314

**Published:** 2025-11-24

**Authors:** Jiawen Xu, Zixuan Xia, Yebao Xia, Ru Zhang, Jianjun Ge

**Affiliations:** 1 Jiangsu Key Laboratory of Robot Sensor and Control Laboratory, School of Instrument Science and Engineering, Southeast University, Nanjing 210096, China; 2 Institute of Biomedical Devices, Southeast University, Suzhou 215163, China; 3 Nanjing Engineer Institute of Aircraft Systems, Nanjing 210096, China

**Keywords:** piezoelectric energy harvesting, zigzag-shaped, multiple piezoelectric transducers, self-powered sensor node

## Abstract

A slender piezoelectric cantilever for energy harvesting features high efficiency but a low operational frequency, which cannot meet the requirements for self-powered transformer monitoring. In this research, a piezoelectric vibration energy harvester with a zigzag root section was proposed. The harvester has a vertically arranged zigzag root section, enabling the capability of integrating multiple piezoelectric transducers as well as increasing the resonant frequency and reducing strain. A linear electromechanical coupling model was formulated. The theoretical and experiential base-movement analysis demonstrates that the zigzag root section of the piezoelectric cantilever beam can effectively reduce the equivalent mass of the system and thereby increase the resonant frequency of the slender beam to 100 Hz, the vibration frequency of a transformer. It was also experimentally shown that the harvester can output 13.54 mW of power. An STM32 microcontroller (MCU) based sensor node with a wireless data transmission module was then designed for evaluations. The piezoelectric harvester with a zigzag root section can successfully support a wireless sensor for transformer monitoring.

## 1. Introduction

Smart transportation, smart cities, and smart grids have employed numerous wireless sensors for sensing and diagnosing [1,2,3,4,5]. Among different types of energy harvesters, piezoelectric cantilever-based vibration energy harvesters (PEH) have received significant attention due to their simplicity and high efficiency [6,7]. At the early stage, the mechanical structures of piezoelectric energy harvesters have been optimized in aspects of thickness ratios of substrate and piezoelectric transducers [8,9,10,11,12]. In addition, the piezoelectric transducer suffers from uneven strain distribution in the longitudinal direction [13]. To obtain enhanced power output of the piezoelectric energy harvester, Baker et al. proposed a trapezoidal beam for uniform strain distribution, yielding twice the power output capability [14]. The concept of strain smoothing was then intensively discussed [13,14,15,16,17].

Considering the fact that ambient vibrations in real applications feature a broad frequency bandwidth, various broadband piezoelectric vibration energy harvesters have been proposed [18]. Mechanisms for the broadband operation include Duffing-type nonlinearity [19,20], segmental stiffness-induced nonlinearity [21,22], internal resonance [23,24,25,26], and multimodal [27]. For instance, PEHs based on a Duffing-type mechanism have been extensively investigated due to their capability to work over a wide frequency range [28,29,30,31,32,33,34,35]. In addition, among the Duffing-type harvesters, the bistable one shows enhanced efficiency, as it vibrates between two potential wells, with enlarged amplitude of displacement and bandwidth [28,29,30]. It was also demonstrated that the inter-well oscillations of a bistable PEH had much better performance than that of a monostable or an intra-well one [34,35]. In addition, PEHs with multiple stable and annual stable potential wells in two-dimensional space can significantly reduce the limitations of the potential barrier under small excitations [36,37,38,39]. More recently, a multi-stable oscillator with programmable nonlinear force has been proposed with extraordinary performances for vibration energy harvesting and vibration isolation [40,41].

With the development of energy harvesting, researchers have focused on vibration energy harvesting across different application scenarios [42,43]. For example, piezoelectric skin-based vibration energy harvesting was proposed for powering sensor nodes on air-conditioners [44]. This is also an attractive option for high-voltage transformers due to the common occurrence of vibrations in transformers [45,46,47]. Vibration energy harvesting allows a continuous power supply that overcomes the limitation of solar energy in cloudy conditions or at night. Although nonlinear broadband energy harvesters are promising, the conventional constant-sinusoidal excitation of a high-voltage transformer would yield optimal power output in a linear system. This is because the transformer has a constant excitation frequency of 100 or 120 Hz due to the fixed operational frequency of 50 or 60 Hz of the power system [45,46,47]. Conventionally, slender piezoelectric cantilevers were chosen as they can generate large strain on the surface, i.e., generating large voltage output through the electromechanical coupling effect of the transducer. However, a slender piezoelectric cantilever would have a resonant frequency of around 10~50 Hz, which is much lower than the operational frequency of a transformer [28,29,30,31,32,33,34,35,36,37,38,39]. Therefore, a lender beam with low resonant frequency would work off-resonance for electrical transformer energy harvesting, with low efficiency. In addition, due to the breakdown strain and voltage constraint, a single piezoelectric transducer has a limited output [13,14,15]. Therefore, two challenges should be overcome to build a highly efficient energy harvester for transformers. Firstly, we need to increase the resonant frequency of a slender piezoelectric cantilever to 100 Hz while maintaining its efficiency for high-frequency operation. In addition, multiple transducers can be introduced to enhance the power output of a PEH for self-powered transformer monitoring.

Notably, zigzag structures are a type of cantilever that makes it feasible to embed multiple piezoelectric transducers. Karami introduced a zigzag structure with multiple piezoelectric components to harvest low-frequency mechanical energy [48,49]. Then, Berdy and Bai designed energy harvesters based upon similar zigzag structures [50,51]. Besides, Abdelmoula investigated the torsion dominant mode of the zigzag harvester and demonstrated its features [52]. Shi claimed that a zigzag harvester has the capability of high-power density [53]. In the previous zigzag harvesters, the cantilevers were usually cut into zigzag shapes and vibrated perpendicular to the cantilever. As these zigzag-shaped structures feature reduced and multiple resonant frequencies, they were usually adopted for low-frequency and broadband vibration energy harvesting.

On the other hand, the transformer has a constant operational frequency within a relatively high frequency range. To power a sensor node for a transformer, an ideal harvester would benefit from enhanced power output and high efficiency at the relatively high operational frequency point. The authors previously investigated magnetic energy harvesting from power transmission systems [54]. However, the proposed harvester has relatively low efficiency. Inspired by zigzag structures, in this study, we propose an upright zigzag-shaped cantilever for vibration energy harvesting in high-voltage transformers. In contrast to previously designed zigzag structures in a 2-dimensional plane, the proposed structure has vertically arranged zigzag cantilevers in 3-dimensional space at the root section of the cantilever, thereby reducing the overall length of the cantilever and increasing resonant frequency. In addition, the vertically arranged zigzag structure would benefit from the torque-induced strain-smoothing effect. It is hypothesized that combining the zigzag configuration and the strain-smoothing effect, we can achieve effective vibration energy harvesting. In addition, a case study was carried out with wireless sensing to evaluate the proposed PEH for transformer monitoring.

The remainder of this paper is organized as follows: the system configuration and model of the zigzag harvester are presented in Section 2. In Section 3, case studies are presented to illustrate the performance of the zigzag energy harvester. In Section 4, experimental studies are presented. Finally, the conclusions are provided in Section 5.

## 2. System Configuration and Modeling

### 2.1. System Configuration

**Figure 1:**
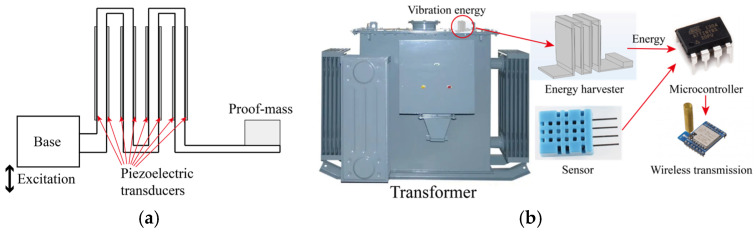
(**a**) Prototype of the piezoelectric upright zigzag energy harvester; (**b**) self-powered sensor node for transformer monitoring.

A prototype of the PEH is plotted in Figure 1. The PEH system is assembled by a vertically arranged zigzag root section and a horizontal cantilever section. The zigzag section has a cantilever folded into a castle shape in the 3-dimensional space. Eight pieces of piezoelectric transducers are glued on the surface of the cantilever in the zigzag section, as shown in Figure 1a. Besides, a proof mass is placed at the end of the horizontal cantilever section. This harvester is subjected to z-direction excitation that is perpendicular to the cantilever section, i.e., parallel to the cantilevers in the zigzag section. Note that the proposed PEH is designed for self-powered sensing on a high-voltage transformer with a vibration frequency of 100 Hz. A wireless sensor node is built for evaluation.

The unique design of the vertically arranged zigzag root section yields several advantages. Firstly, the zigzag section is assembled in the root section of the cantilever. This is because the bending moment within the cantilever would accumulate gradually in the longitudinal direction of the cantilever system, i.e., the root section of the cantilever would have the largest strain distribution within the system. Therefore, integrating a zigzag section within the root section of the cantilever would make full use of the accumulated bending moment. In addition, multiple piezoelectric transducers were attached to the zigzag section of the cantilever to enhance the power output capability of the system. Moreover, with such a configuration, the reduced length of the device can effectively reduce the equivalent mass and thereby increase the resonant frequency. Notably, unlike the conventional zigzag structure in a 2-dimensional space, the proposed folded structure would not suffer from twisting-induced uneven strain in the width direction.

Indeed, increasing the thickness of the beam would also increase the resonant frequency. On the other hand, a thick beam has a large shear strain within the beam, as illustrated by Timoshenko beam theory. Note that the piezoelectric transducers are typically attached to a beam using epoxy resin, which has a Young’s Modulus hundreds of times smaller than that of a spring steel beam. Hence, a large shear strain within the composite piezoelectric beam would prohibit the transmission of bending moment from the beam to the piezoelectric transducers, yielding reduced power output capability. Here, we focus on the optimization of the configuration of a slender cantilever for a new mechanism to increase the resonant frequency with the capability of integrating multiple piezoelectric transducers and build a case study to demonstrate its performance in applications. The proposed design can potentially be applied in the field of energy harvesting in transformer substations.

### 2.2. Mathematical Modeling

**Figure 2:**
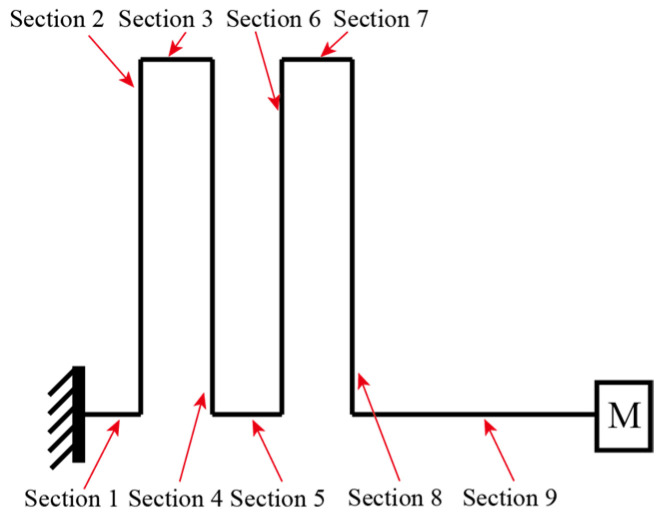
Model of the upright zigzag vibration energy harvester.

A model of the piezoelectric cantilever is formulated. Euler cantilever theory is employed to solve the mode shape of the zigzag cantilever. We divide the cantilever into nine sections, each of which is marked in Figure 2. The piezoelectric transducers are attached to the two surfaces of the 2nd, 4th, 6th, and 8th sections. We assume a uniform equivalent Young’s Modulus in each section $EI_{i}$. The Equation for the sections with and without a piezoelectric transducer attached can be found in Refs. [55,56,57]. In the following analysis, the *i*-th section’s length is denoted as li. $m_{i}$ is the mass density of the cantilever of unit length in the *i*-th section. As similar modeling of a uniform piezoelectric cantilever has been intensively discussed in the literature, only the key equations are presented here.

The modal displacement for the undamped modal of the *i*-th cantilever is given [54]:

(1) $$w_{i}\left(x_{i},t\right)=\phi_{i}\left(x_{i}\right)q\left(t\right)$$

where $w_{i}\left(x_{i},t\right)$ stands for the displacement of the *i*-th cantilever in the bending direction at time *t*. As the proposed PEH works at a single frequency point in the target scenario, the first bending mode of the system is evaluated. In addition, $\phi_{i}\left(x\right)$ denotes the mode shape and q(t) stands for modal displacement. We let the mode shapes of the cantilever be represented as $\phi_{i}(x_{i})$ (*i* = 1, 2,…, 9) for the cantilever sections [55],

(2) $$\phi_{i}(x_{i})=A_{i}\cos k_{i}x_{i}+B_{i}\sin k_{i}x_{i}+C_{i}\cos hk_{i}x_{i}+D_{i}\sin hk_{i}x_{i}$$

where *A_i_*, *B_i_*, *C_i_*, and *D_i_* are coefficients of the mode shape functions of the *i*-th section. The eigenvalues are $k_{i}=\sqrt[{4}]{\omega^{2}\frac{\rho_{i}A_{i}}{EI_{eqi}}}$. As the transformer is working on a single frequency point, the harvester is working on the first bending mode. The boundary conditions at the fixed end of section 1 are given

(3) $$w_{1}(0,t)=0,\;{\left.\frac{\partial w_{2}(x_{1},t)}{\partial x_{1}}\right|}_{x_{1}=0}=0$$

The remaining boundary conditions of the first section and those between the first and second sections are given, respectively,

(4) $$w_{2}(0,t)=0,\;{\left.\frac{\partial w_{1}(x_{1},t)}{\partial x_{1}}\right|}_{x_{1}=l_{1}}={\left.\frac{\partial w_{2}(x_{2},t)}{\partial x_{2}}\right|}_{x_{1}=0},\;\;{\left.EI_{1}\frac{\partial^{2}w_{1}(x_{1},t)}{\partial^{2}x_{1}}\right|}_{x_{1}=l_{1}}=EI_{2}{\left.\frac{\partial^{2}w_{2}(x_{2},t)}{\partial^{2}x_{2}}\right|}_{x_{2}=0},\;\;{\left.EI_{1}\frac{\partial^{3}w_{1}(x_{1},t)}{\partial^{3}x_{1}}\right|}_{x_{1}=l_{1}}=\left(M+\sum_{i=2}^{9}m_{i}l_{i}\right){\left.\frac{\partial^{2}w_{1}(x_{1},t)}{\partial t^{2}}\right|}_{x_{1}=l_{1}},$$

Besides, the continuity relations at the joint between the *i*-th and (*i* + 1)-th sections of the proposed zigzag cantilever are given, respectively,

(5) $$w_{i-1}(l_{i-1},t)=w_{i+1}(0,t),\;{\left.\frac{\partial w_{i}(x_{i},t)}{\partial x_{i}}\right|}_{x_{i}=l_{i}}={\left.\frac{\partial w_{i+1}(x_{i+1},t)}{\partial x_{i+1}}\right|}_{x_{i+1}=0},\;\;{\left.EI_{i}\frac{\partial^{2}w_{i}(x_{i},t)}{\partial^{2}x_{i}}\right|}_{x_{i}=l_{i}}=EI_{i+1}{\left.\frac{\partial^{2}w_{i+1}(x_{i+1},t)}{\partial^{2}x_{i+1}}\right|}_{x_{i+1}=0},\;\;{\left.EI_{i}\frac{\partial^{3}w_{i}(x_{i},t)}{\partial^{3}x_{i}}\right|}_{x_{i}=l_{i}}=\left(M+\sum_{j=i+1}^{9}m_{j}l_{j}\right){\left.\frac{\partial^{2}w_{i}(x_{i},t)}{\partial t^{2}}\right|}_{x_{i}=l_{i}}$$

Consider a proof-mass *M* with inertial *J* attached that can be found; the continuity relations at the free end of section 9 are given:

(6) $${\left.EI_{9}\frac{\partial^{3}w_{9}(x_{9},t)}{\partial^{3}x_{9}}\right|}_{x_{9}=l_{9}}=M{\left.\frac{\partial^{2}w_{9}(x_{9},t)}{\partial t^{2}}\right|}_{x_{9}=l_{9}},\;{\left.EI_{9}\frac{\partial^{2}w_{9}(x_{9},t)}{\partial^{2}x_{9}}\right|}_{x_{9}=l_{9}}={\left.J\frac{\partial^{3}w_{9}(x_{9},t)}{\partial t^{2}\partial x_{9}}\right|}_{x_{9}=l_{9}}$$

Grouping Equations (4)–(6) together, we obtain the eigenvalue problem regarding the coefficients of the 1st to 9th segments of the piezoelectric cantilever

(7) $$d={\left[\begin{array}{cccccccccccccc} A_{1} & B_{1} & C_{1} & D_{1} & ... & A_{i} & B_{i} & C_{i} & D_{i} & ... & A_{9} & B_{9} & C_{9} & D_{9} \end{array}\right]}^{T}$$

We have

(8) $$K_{1}\left(\lambda_{1}...\lambda_{i}...\lambda_{9}\right)d=0$$

The matrix ***K***_1_ is not listed here due to its large size. The characteristic equation of the cantilever system with zigzag root-section can be solved by letting the determinant of ***K***_1_ be zero numerically. In addition, the mode shape $\phi_{i}(x_{i})$ of different sections of the proposed PEH can be solved by evaluating the eigenvector d.

The governing equations of the zigzag harvester can be obtained considering the electromechanical coupling as [52,53,54]:

(9) $$\frac{d^{2}q(t)}{dt^{2}}+2\varsigma \omega_{1}\frac{dq(t)}{dt}+\omega_{1}^{2}q(t)+\sum_{k=1}^{8}\chi_{k}V_{k}(t)=F\left(t\right)$$

where ς is the viscous modal damping ratio, $\chi_{i}$ and $V_{k}(t)$ is the electromechanical coupling coefficient and voltage output of the *k*-th piezoelectric transducer attached on the two surfaces of the 2nd, 4th, 6th, and 8th sections, respectively. The $\chi_{i}$ can be obtained through the calculation using the solved modal shapes [52]. Besides, $F\left(t\right)$ is the modal force under sinusoidal base excitation $a\left(t\right)$. The characteristic of the PEH in the electrical domain is given

(10) $$\frac{1}{R}+\frac{\varepsilon_{33}^{s}bl_{i}}{h_{p}}\frac{dV(t)}{dt}=-e_{31}b\frac{h_{p}+h_{b}}{2}\int_{0}^{l_{pi}}\frac{\partial^{3}w_{i}(x_{i},t)}{\partial^{3}x_{i}}dx_{i}$$

where $\varepsilon_{33}^{s}$ is the dielectric constant under constant stress, *b* and *h_p_* are the width and thickness of the piezoelectric transducer, $e_{31}$ is the piezoelectric coefficient, and $h_{b}$ is the thickness of the substrate cantilever. The solution of the electro-mechanical coupled system can be obtained assuming harmonic responses of the displacement and voltage outputs.

## 3. Case Studies

**Figure 3:**
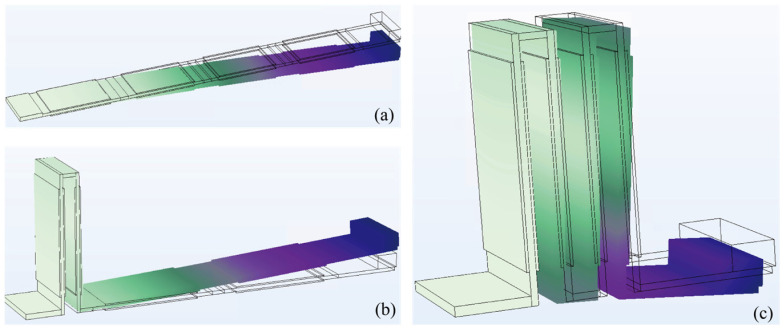
The first bending mode of a piezoelectric cantilever (**a**) Case 1: without zigzag section, (**b**) Case 2: with one folding, and (**c**) Case 3: with three foldings.

To demonstrate the key advantage of the integrated zigzag section, the vibration mode and responses of the piezoelectric transducers are first analyzed. In this section, we consider three cases: Case 1 has no folding, as shown in Figure 3a, representing a conventional piezoelectric cantilever. Case 2 has one folding at the root of the beam, as shown in Figure 3b. Case 3 has three folding at the root of the cantilever, as shown in Figure 3c. It is worth mentioning that all three cases have eight pieces of 30 × 20 × 0.2 mm^3^ piezoelectric transducers attached to the top and bottom surfaces of the substrate. Moreover, a same-size proof mass was attached to the free end of the conventional cantilevers. In the following analysis, Rayleigh damping was adopted as the performance of the harvesters is evaluated over a large frequency range.

Here, we designed the three cases with the same fully extended beam length of 201 mm. In other words, Case 1 without folding has the longest physical length with the lowest resonant frequency of 22.5 Hz. Besides, the proposed harvester with three folding has the most compact physical size with the highest resonant frequency of 100 Hz, due to the compactness introduced by the zigzag configuration, as illustrated in Table 1. This is because the piezoelectric cantilever is rolled up in the zigzag section, thereby reducing the overall lumped mass of the system. 

**Figure 4:**
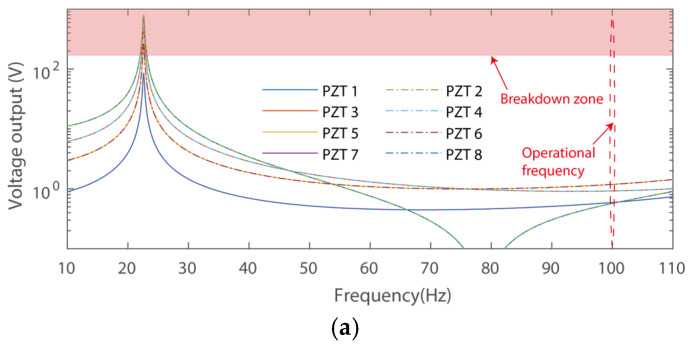

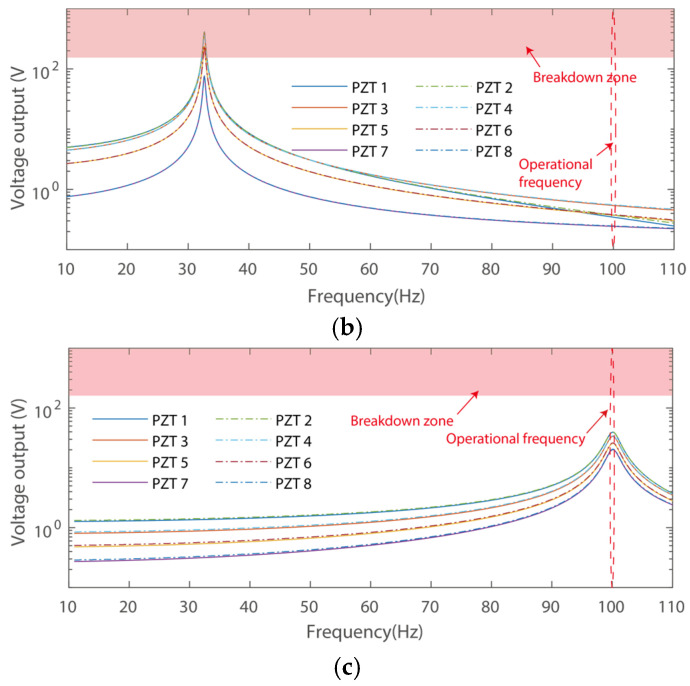
Open circuit voltage output of: (**a**) Case 1: without folding, (**b**) Case 2: with one folding, and (**c**) Case 3: with three folding. (Red regions: high voltage breakdown).

Analysis of the voltage and power output performance for the three cases was then carried out to demonstrate the features of the cantilever with a zigzag root section and the conventional PEH, as shown in Figure 4.

Figure 4 presents the voltage outputs of the eight piezoelectric transducers for the three harvesters, respectively. It can be obtained that the maximum voltage outputs of the harvesters are 796 V, 410 V, and 40 V, respectively, at their resonant frequencies. Notably, the power output is also hinged upon the operational frequencies, as would be illustrated in the following discussion. In addition, the maximum voltage outputs are 1.2 V, 0.5 V, and 40 V, respectively, at the target operational frequency of 100 Hz. In other words, although the conventional design has large voltage outputs at its resonant frequency, it has minor outputs and cannot meet the requirements of a power supply under excitation of 100 Hz. Indeed, increasing the thickness can raise the resonant frequency. However, layers between the substrate and the piezoelectric transducer would bear a large shear strain, yielding significantly reduced efficiency. Besides, shrinking the length of the piezoelectric cantilever may also increase its resonance. However, a cantilever with reduced length cannot support integration of the eight piezoelectric transducers. In addition, the proposed design features the advantage of compactness for the application scenario of the high-voltage transformer, as the length of the PEH can be shortened within 40 mm.

It can also be obtained that the voltage outputs of the harvester in Case 1 have large variations. In particular, the lowest voltage output is 10.7% of that of the maximum one. The phenomenon can be attributed to the fact that enlarging the length of the beam would yield a large lumped mass, i.e., reduced resonant frequency, with strain concentration at the fixed end. Besides, the PEH with three folding has the lowest voltage output, 51.4% of that of the maximum one, i.e., the proposed harvester has a more smoothed voltage output from the piezoelectric transducers. It is also worth mentioning that the piezoelectric transducer would break down under high voltage. For instance, the PZT-5H has a typical Coercive Field of breakdown of 8 kV/cm. In such a case, the piezoelectric transducers adopted in this study would bear a maximum voltage of 160 V only, i.e., most of the piezoelectric transducers would break down in Cases 1 and 2 at their resonances, respectively.

**Figure 5:**
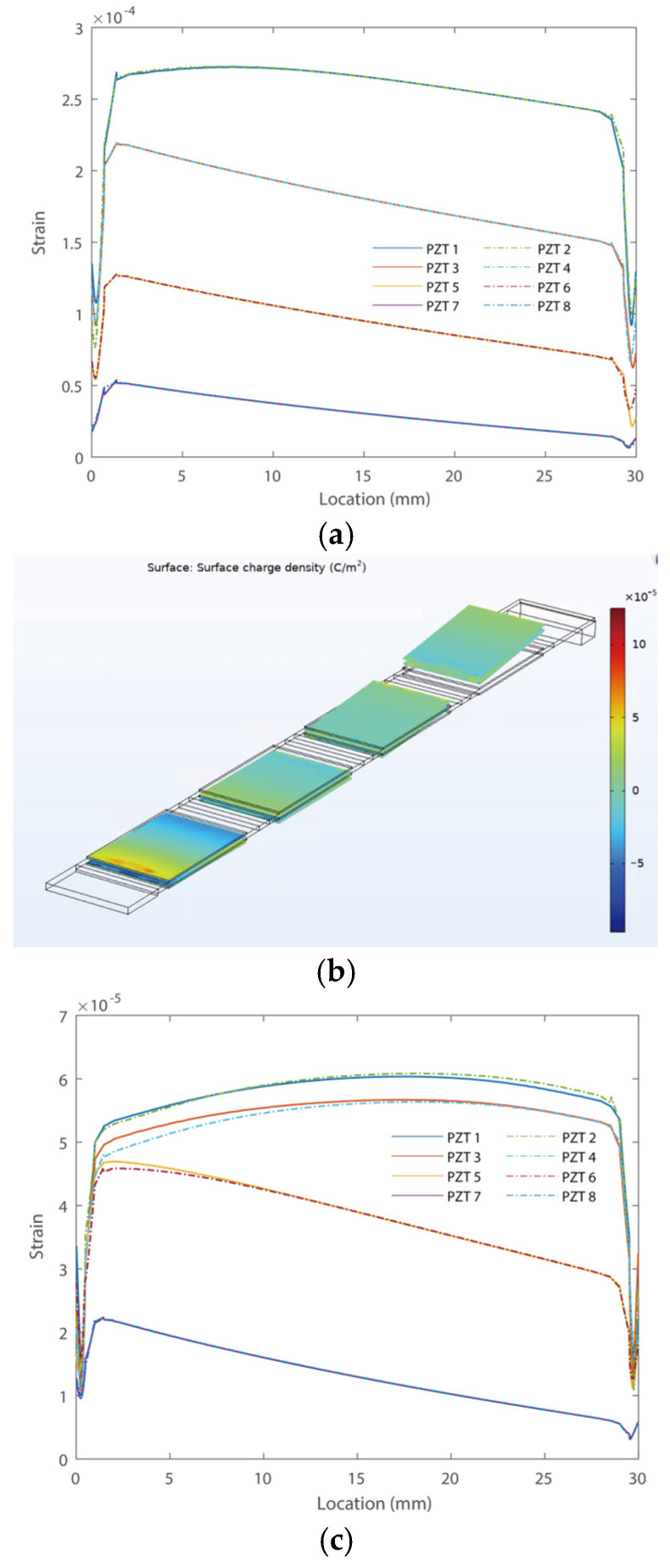

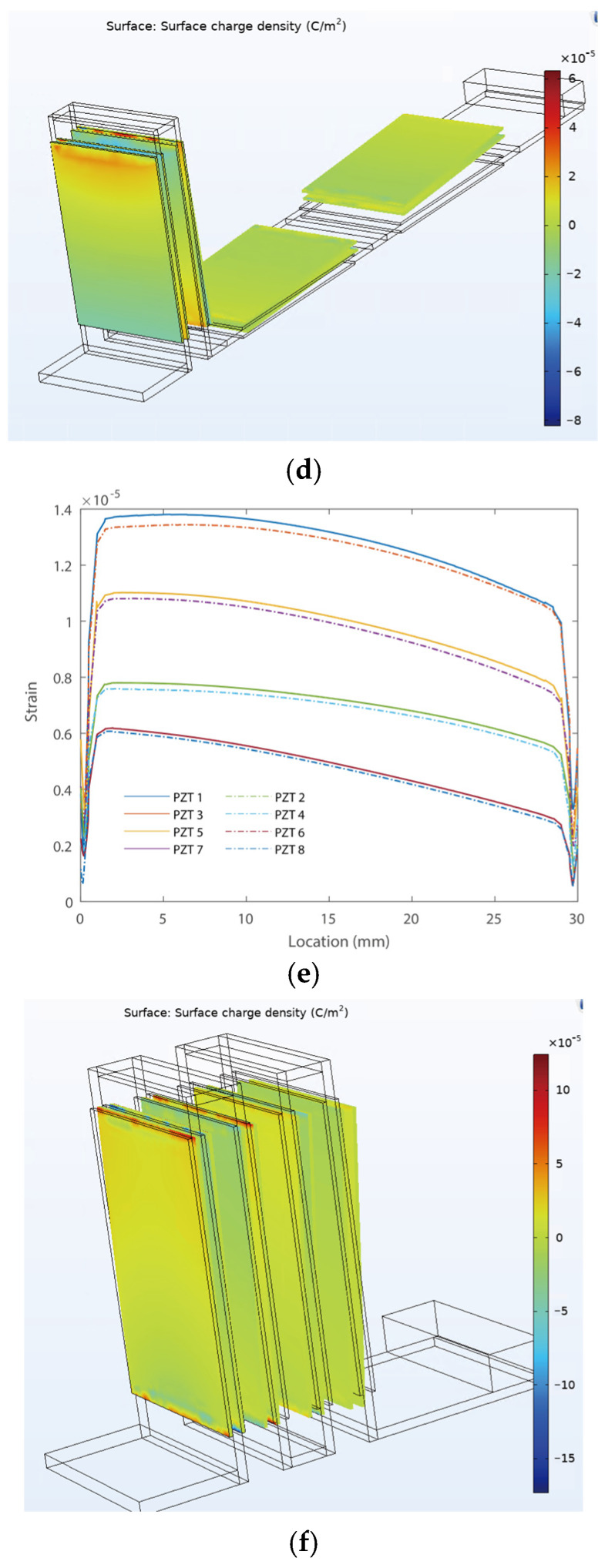
(**a**) Case 1: strain without folding, (**b**) Case 1: surface charge distribution without folding, (**c**) Case 2: strain with one folding, (**d**) Case 2: surface charge distribution with one folding, (**e**) Case 3: strain with three folding and (**f**) Case 3: surface charge distribution with three folding along the piezoelectric transducers.

Figure 5 shows the strain and charge density distributions along the piezoelectric transducers of the first bending modes of the PEHs, respectively. The strain and charge density distributions were evaluated under the resonant frequencies of each PEH in the three cases. It can be obtained that the zigzag cantilever has a pure bending motion in the longitudinal direction of the cantilever, i.e., it does not have a twisting motion as the conventional two-dimensional zigzag designs, which may reduce the overall efficiency of the harvester. In addition, the PEH in Case 1 without a zigzag section has a strain up to 8.1 × 10^−4^, as shown in Figure 5a. Notably, the strain is extremely large as the piezoelectric transducer of PZT-5H would fragment with a strain of around 3 × 10^−3^. In other words, the piezoelectric transducers in Case 1 would easily break down electrically or fragment physically. Besides, the strain distribution of PZT 1 and 2 is almost the same, as they are attached symmetrically to the top and bottom of the substrate. In addition, all piezoelectric transducers have a gradient strain distribution due to gradient bending torque. The piezoelectric transducers of PEH in Case 2 have a much smaller strain, as shown in Figure 5c. For instance, the maximum strain in this case is 6.1 × 10^−5^, which is more than one order smaller than that in Case 1. It can also be obtained that the piezoelectric transducers 1~4 have more smoothed strain distributions than those of the piezoelectric transducers 5~8. This is because the piezoelectric transducers 1~4 are attached to the zigzag section, configured in the vertical direction, with little change in excitation-induced bending torque.

The piezoelectric transducers in the PEH in Case 3 bear the smallest strain in all three cases, with a maximum value of 1.38 × 10^−5^, as shown in Figure 5e. Smoothed strain distributions can also be obtained in the proposed zigzag one. This is because the bending moment would be transmitted in the bending direction and accumulate at the root section of the cantilever, thereby creating a smoothed strain distribution of the proposed harvester. Besides, the strain distributions of the two transducers attached to the two surfaces of the same cantilever are almost the same in the proposed zigzag one. Moreover, the transducers in the vicinity of the fixed end have a higher amplitude than those at the free end. The gradients of the strain distributions of the transducers at the free end show an increasing trend compared with those around the fixed end. Notably, conventional zigzag structures in the two-dimensional plane would transmit torque perpendicular to the bending direction of the cantilever, which yields the twisting motion of the cantilever. Thus, the conventional zigzag structure may experience both positive and negative strain distribution in one piezoelectric transducer with reduced efficiency. The proposed zigzag harvester in three-dimensional space has only the bending moment applied along the longitudinal direction, thereby yielding better performance.

**Figure 6:**
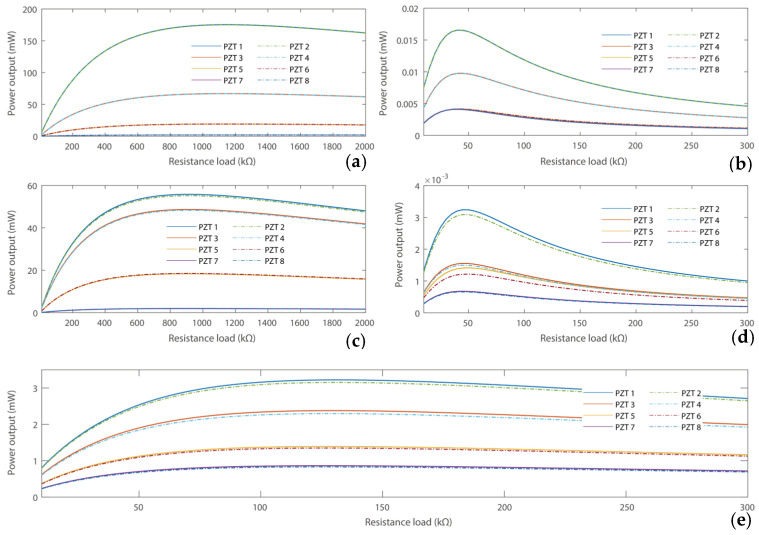
Power output performances under different resistance loads (**a**) Case 1 at resonant frequency; (**b**) Case 1 at 100 Hz; (**c**) Case 2 at resonant frequency; (**d**) Case 2 at 100 Hz; and (**e**) Case 3 at resonant frequency of 100 Hz.

We then evaluate the power output capability of the proposed PEH using resistance loads. Here, two scenarios are considered: firstly, we calculated the power output of the PEHs at their resonances, respectively. Secondly, the power output performances at the operational frequency of 100 Hz are calculated, as shown in Figure 6.

The power output performances of the PEHs in the three scenarios are shown in Figure 6, which are evaluated at the resonant frequencies of each PEH and the operational frequency of 100 Hz, respectively. It can be obtained that the optimal resistance loads for the three PEHs differ in the resonant condition due to the different capacitance impedances of the piezoelectric transducers. Typically, the optimal resistance load is $R_{opt}=\frac{1}{C_{p}\omega }$ [58]. Besides, the optimal matching resistance loads are almost the same at the operational frequency of 100 Hz. It can also be obtained that the power outputs of piezoelectric transducers in the PEH in Case 1 at resonance vary significantly with each other due to the gradient strain distributions and voltage outputs, as illustrated in Figure 3 and Figure 4. Besides, the power outputs of the PEH in Case 3 are close to each other at resonances. For comparison, the total power output performances of the harvesters in the three cases are listed in Table 2.

Table 2 presents the total power outputs of the three PEHs at resonant frequencies and 100 Hz, respectively. It can be obtained that the PEH in Case 1 has the maximum power output of 526.48 mW at its resonant frequency of 22.5 Hz. However, as the targeted operational frequency of the Transformer substation is 100 Hz, the power output drops significantly to 0.069 mW at this frequency point. The PEH in Case 2 has a power output of 248.38 mW at its resonant frequency of 32.4 Hz. However, as the targeted operational frequency of the Transformer substation is 100 Hz, the power output drops significantly to 0.013 mW at this frequency point. Besides, as the resonant frequency of the PEH with zigzag root section is raised to 100 Hz, the total power output of the one in Case 3 has been raised to 15.47 mW in this scenario, which exceeds the performances of PEHs in Cases 1 and 2 at the operational frequency. The results from the three cases confirm the advantages of the proposed harvester. In particular, integrating a zigzag root section can effectively increase the resonance frequency of the harvester while yielding large power outputs at the target operational frequency with a capability of powering wireless sensor nodes.

## 4. Experimental Results and Discussion

### 4.1. Experimental Setup

**Figure 7:**
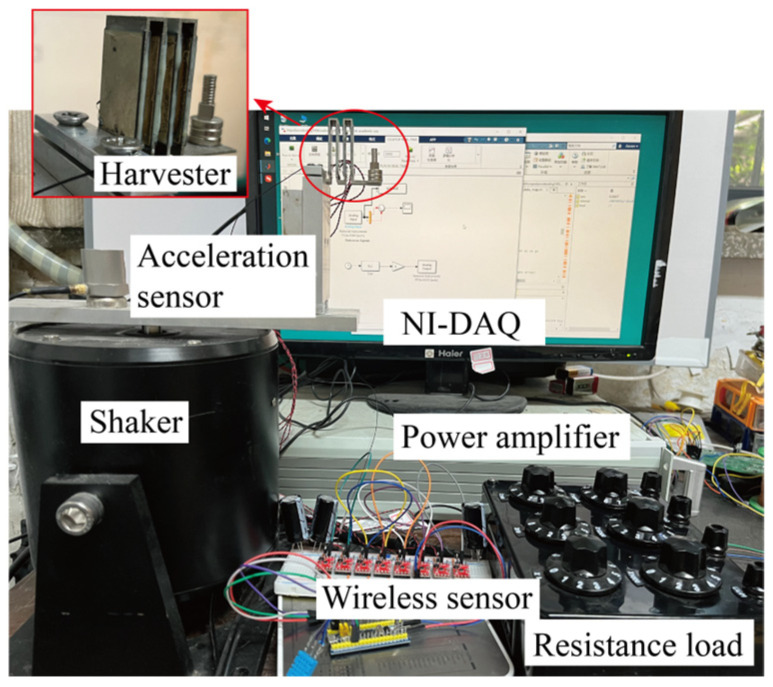
Experimental set-up.

Experimental studies were carried out to evaluate the performance of the proposed PEH with a zigzag root section. Figure 7 illustrates the experimental setup. The cantilever consists of a zigzag section and a conventional cantilever section. The 2nd, 4th, 6th, and 8th sections have dimensions of 38 × 20 × 1 mm^3^. Sections 3, 5, and 6 have dimensions of 6 × 20 × 1 mm^3^. The 9th section has a dimension of 20 × 20 × 1 mm^3^. Eight 30 × 20 × 0.2 mm^3^ piezoelectric transducers were attached to the surfaces of the cantilever in the zigzag section, respectively. The piezoelectric device has a dimension of 48 × 20 × 11 mm^3^. The PZT-5 piezoelectric transducers were purchased from Jiayeda, Co., Ltd., Changde, China. The piezoelectric materials had a Young’s Modulus of 106 GPa and a charge coefficient of d_31_ = −171 pC/N. Besides, a proof mass was attached using multiple permanent magnets with careful tuning of the resonant frequency around 100 Hz. The proof mass was assembled with permanent magnet blocks. The permanent magnets provide an attractive force to fix the location of each additional magnet during the process of resonant frequency adjustment. A shaker provides excitations in the *z*-direction. The experiment was conducted under constant excitations, which were measured by an accelerometer (CT1020LC, CHENGTEC, Shanghai, China). Besides, the voltage outputs were measured in an open circuit condition as the probes have input impedances of 100 MΩ. All the data was recorded by a data acquisition device (PCIe6343, National Instrument, Austin, TX, USA) at a sampling frequency of 20 kHz. The root-mean-square (RMS) values of the outputs were calculated. The amplitude of the base movement was. In addition, the gravitational constant g = 9.8 m/s^2^ is chosen.

### 4.2. Output Performance

We then measure the output performance of the proposed PEH with a zigzag root section. It is worth noticing that a power transformer features excitations at 100 Hz. Hence, the outputs of the proposed PEH were evaluated in the vicinity of this frequency point. Here, the voltage and power outputs of all eight piezoelectric transducers were evaluated independently for conceptual illustrations.

**Figure 8:**
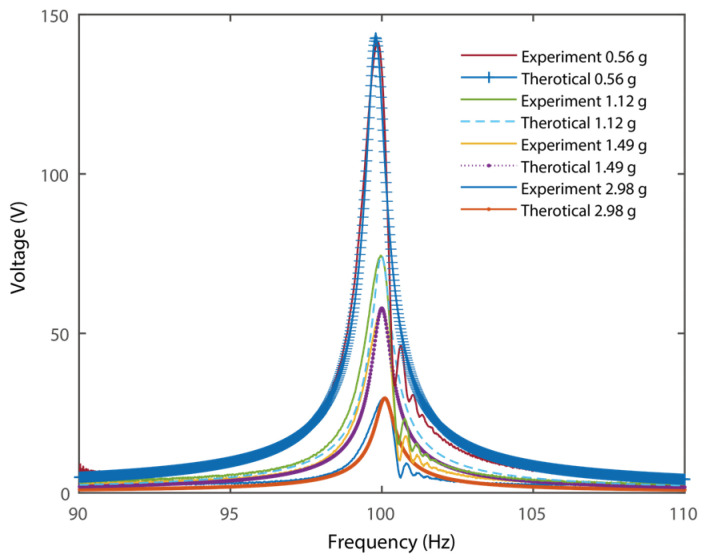
Theoretical and experimental open circuit voltage output of the PZT at the fixed end.

Figure 8 shows the theoretical and measured voltage output of the PZT attached to Section 2 (at the fixed end) under excitations with different amplitudes. It can be obtained that the proposed PEH with zigzag root sections outputs 142 V under 2.98 g excitation. Typically, the real acceleration values of a transformer substation were 0.3~4 g in real applications [45,46,47]. Therefore, the acceleration values of excitations were chosen in this range for evaluation. The voltage outputs increase with the rise of excitations. As the zigzag energy harvester is essentially a linear system, the system dynamics are much simpler than those of a nonlinear one. Nevertheless, the optimal performance at 100 Hz with a compact configuration can be fully verified. Besides, the experimental results agree well with the theoretical analysis.

**Figure 9:**
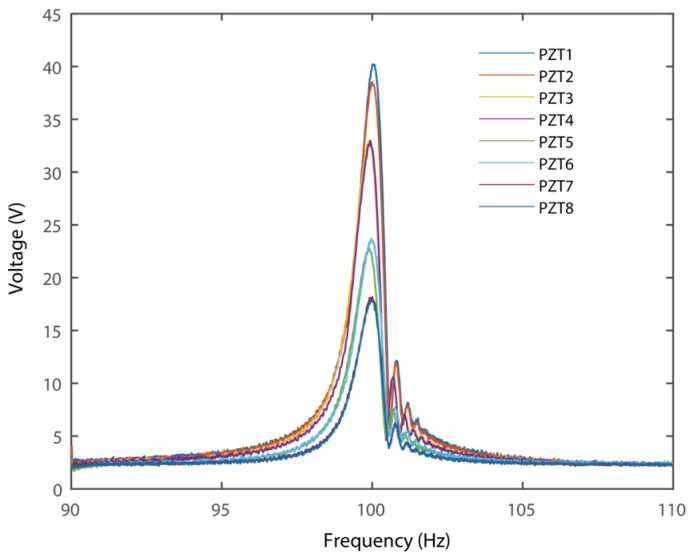
Experimental open circuit voltage output of all the piezoelectric transducers.

Note that the harvester contains eight transducers; the voltage outputs of each PZT were compared in Figure 9. All the data were measured under excitations of 0.75 g. It can be obtained that the piezoelectric transducer has a much larger voltage output than the one attached at the free end. This phenomenon can be attributed to reduced strain within the transducers attached to the free end, as previously demonstrated in Figure 5. Besides, the transducers attached to the two sides of the same beam section have similar voltage output. It follows the trend of the strain distributions as demonstrated in Figure 5.

**Figure 10:**
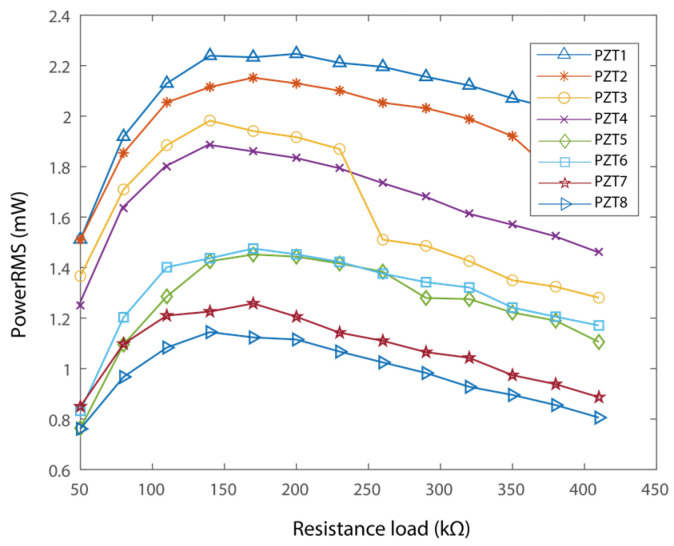
Experimental power output performance.

To measure the power outputs, each of the piezoelectric transducers is connected to independent resistance loads [57,58,59,60]. The resulting power outputs of each piezoelectric transducer are shown in Figure 10. Here, the best match load for each PZT is a function of the resistance loads. It is worth noticing that each of the piezoelectric transducers has slightly different parameters, hence they have different best matching loads. In particular, the best loads are 200 kΩ, 170 kΩ, 140 kΩ, 140 kΩ, 170 kΩ, 170 kΩ, 170 kΩ, and 140 kΩ, respectively, as shown in Figure 10. The output power was 2.25 mW, 2.1 mW, 1.95 mW, 1.9 mW, 1.5 mW, 1.45 mW, 1.25 mW, and 1.14 mW, receptively. A similar trend can be obtained in that the transducer attached at the fixed end would output more energy than that at the free end. In addition, the power output of the proposed PEH sums up to 13.54 mW in total. The resistance loads were connected to estimate the power output capability. Connecting the piezoelectric sheets to voltage-rectifying bridges and capacitors may reduce the output power [59,60]. Nevertheless, the power output of 13.54 mW would be capable of supporting intermittent operation of a sensor node.

### 4.3. Self-Powered Sensing

**Figure 11:**
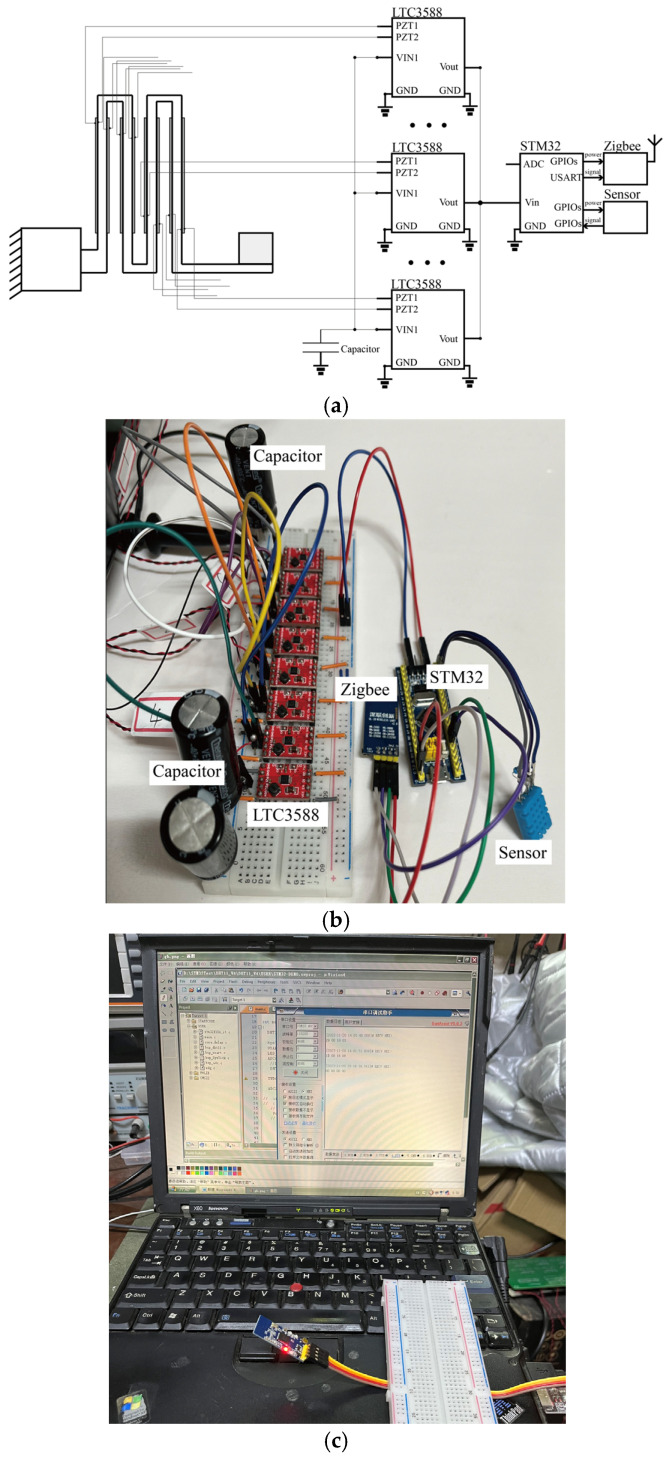
(**a**) Diagram and (**b**) experimental platform of the wireless sensing node and (**c**) host computer.

As the PEH with zigzag root section is designed for self-powered sensing, a sensor node was built, and a case study was carried out. The sensor node has an STM32F103 MCU (STMicoelectronics, Geneva, Switzerland), purchased from STMicroelectronics, a humidity and temperature sensor, a Zigbee wireless module and eight LTC-3588 regulators (Linear Technology, Milpitas, CA, USA), purchased from Linear Technology, that are independently connected to each PZT. Typically, the Zigbee wireless data transmission modules can communicate with each other up to 250 m outdoors. In the experiment, the receiver was placed in the vicinity of the wireless sensor node. The regulated electrical energy is then stored in three 4700 μF capacitors to power the node, as shown in Figure 11. It is worth noticing that the wireless module would consume most of the energy. To reduce the power consumption of the node, we employ multiple GPIOs to provide the electrical energy to the wireless module. In other words, the wireless module would be completely shut down when the output of the GPIOs is set to zero.

**Figure 12:**
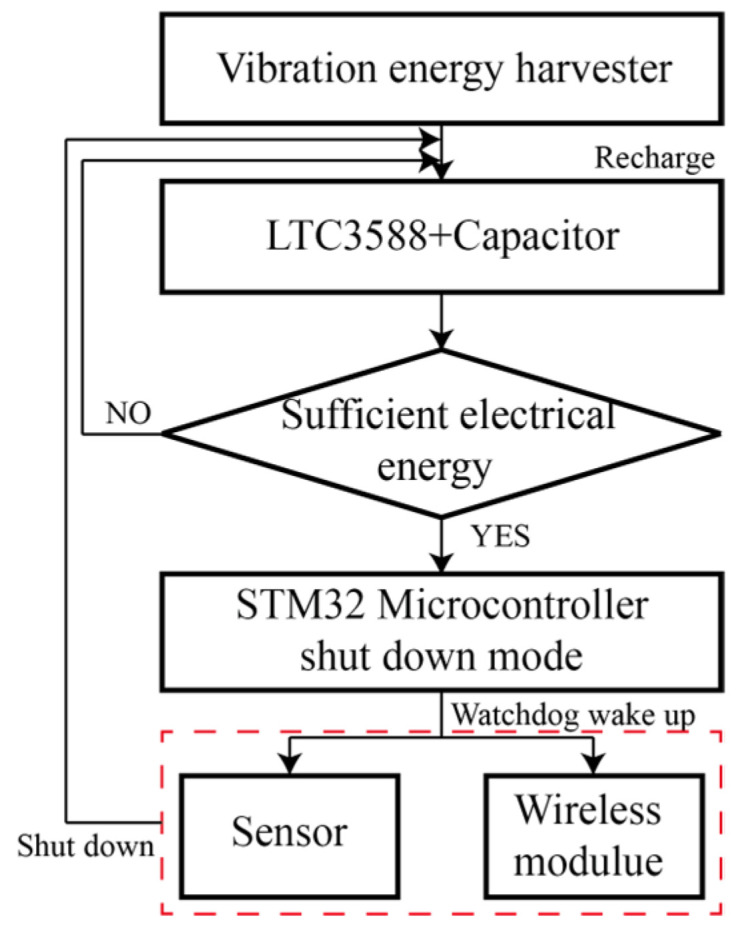
Control algorithm of the wireless sensor node.

The control algorithm is presented in Figure 12. The MCU is shut down to reserve electrical energy most of the time. In addition, the MCU is woken up by a Watchdog at a fixed time interval. When there is sufficient energy in the capacitor, the MCU would then power the sensor and wireless modules. Besides, multiple time delay functions were integrated for the initialization and operation of the sensing and wireless modules to avoid errors.

**Figure 13:**
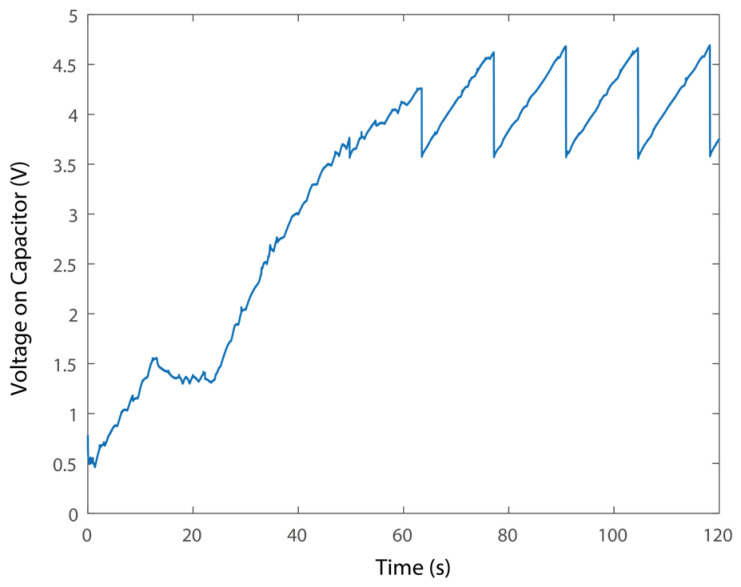
Voltage on the capacitors.

To evaluate the power supply performance of the PEH system, the voltages in the time domain on the capacitors are presented in Figure 13. In particular, the PEH takes about 60 s to collect sufficient energy to power the sensor node for the first time. Besides, it takes about 13 seconds to recharge the capacitor and support the sensor node. The recharging time would be sufficiently short for the regular transformer station monitoring. The voltage drops from 4.679 to 3.569 V during one cycle of wireless sensing, i.e., the wireless sensor node would consume 0.065 J per cycle. In addition, integrating larger capacitors would provide more energy for sensing.

**Figure 14:**
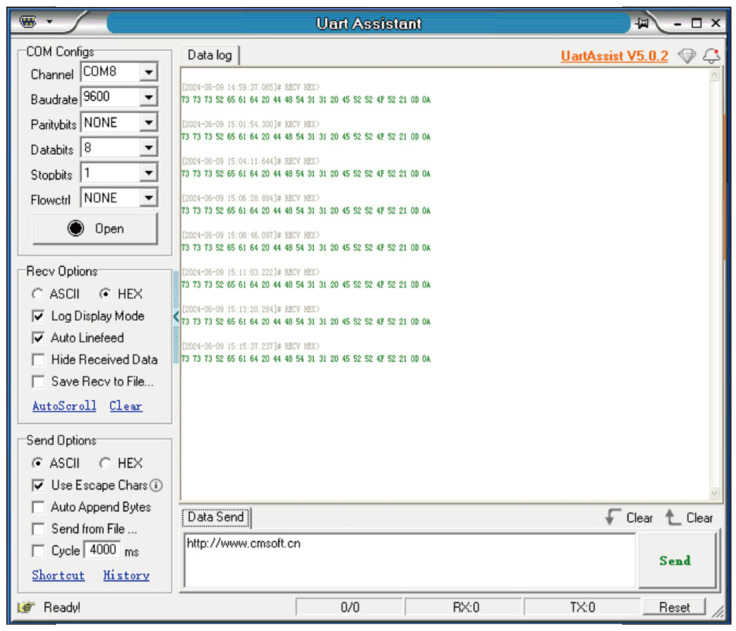
Data received by the host computer.

The data obtained by the self-powered sensor node was transmitted to the host computer, as shown in Figure 14. It is confirmed that the data can be obtained and transmitted successfully without external batteries. Notably, the sensor node with the STM32 MCU is a fully functional unit that can be utilized for edge computing and control. Optimize the operational algorithm may further reduce the power consumption. In addition, optimization can be carried out for enhanced power output of the PEH, including shaping, size, and number of piezoelectric transducers, etc.

## 5. Conclusions

In this research, we proposed a piezoelectric vibration energy harvester with a zigzag root section. A vertically arranged zigzag section was integrated into a slender piezoelectric cantilever. The zigzag section provides the capability of integrating multiple piezoelectric transducers for enhanced power output capability while increasing the resonant frequency for the application on a transformer. We demonstrated analytically and experimentally that the proposed harvester could effectively harvest vibration energy in the environment of a transformer with a power output capability of 13.54 mW under 1.5 g excitation. A wireless sensor node was designed and integrated for the validation of self-powered sensing. The proposed system has potential applications for vibration energy-harvesting-powered wireless sensing in various scenarios.

## Figures and Tables

**Table 1:** Comparison of resonant frequencies.

	Physical Length in Longitude Direction	Fully Extended Length	Resonant Frequency
Case 1	188 mm	188 mm	22.5 Hz
Case 2	112 mm	188 mm	32.4 Hz
Case 3	36 mm	188 mm	100 Hz

**Table 2:** Comparison of total power outputs.

	Power Output at Resonances (mW)	Power Output at 100 Hz (mW)
Case 1	526.48 (break down)	0.069
Case 2	248.38 (break down)	0.013
Case 3	15.47	15.47

## Data Availability

The data presented in this study are available on request from the corresponding author, the data are not publicly available due to privacy or ethical restrictions.
